# Efficacy of stem cell therapy for diabetic kidney disease: a systematic review and meta-analysis

**DOI:** 10.3389/fmed.2025.1601900

**Published:** 2025-09-01

**Authors:** Hongyu Du, Chen Xie, Yiqin Yuan, Yun Luo, Jinguo Cao, Zhihai Li, Jiayi Yuan, Wei Li

**Affiliations:** School of Rehabilitation Medicine, Gannan Medical University, Ganzhou, Jiangxi, China

**Keywords:** stem cells, diabetic nephropathy, clinical trial, meta-analysis, systematic review, randomized controlled trials

## Abstract

**Background/objectives:**

Animal studies have demonstrated the ability of stem cell therapy (SCT) to treat diabetic kidney disease (DKD). However, the efficacy of SCT in patients with DKD remain unclear. This systematic review and meta-analysis aimed to investigate the efficacy of SCT in patients with DKD.

**Methods:**

A comprehensive and systematic literature search was conducted using PubMed, EMBASE, Cochrane Library, and Web of Science to identify articles on SCT for DKD published up to March 2024. RevMan V.5.4 software was used for statistical analysis.

**Results:**

We identified four studies that included 90 participants, 53 (58%) of whom underwent SCT. SCT improved estimated glomerular filtration rate (eGFR) [mean difference (MD) = 0.41, 95% confidence interval (CI): 0.08–0.74; *p* = 0.02], serum creatinine (SCr) reduction (standardized MD = −0.65, 95% CI: −1.19 to −0.1, *p* = 0.02), and microalbuminuria (MAU) (MD = −32.10, 95% CI: −55.26–8.94; *p* = 0.007) compared to the control group, but did not improve urine microalbumin/creatinine ratio (UACR) (MD = −63.36, 95% CI: −194.52–67.79, *p* = 0.56) or blood sugar (MD = 0.49, 95% CI: 4.16–2.01, *p* = 0.49). Adverse events (AEs) were common (67 events in 60 SCT subjects vs. 35 in 28 controls), with urinary system AEs occurring exclusively in the SCT group and nervous system AEs markedly higher.

**Conclusion:**

SCT can effectively improve eGFR and SCr levels by lowering the MAU but cannot improve UACR and blood sugar levels.

## 1 Introduction

Diabetes mellitus (DM) is a chronic disease that poses a significant global public health challenge owing to its high incidence rate (1–6). Diabetic kidney disease (DKD) refers to the progressive deterioration of kidney function in patients with chronic type 1 (T1DM) or type 2 diabetes mellitus (T2DM) (7), representing one of the most serious microvascular complications of diabetes (8). Approximately one-third of patients with T1DM and half of patients with T2DM develop DKD (9). Moreover, DKD accounts for 30 to 50% of end-stage renal disease (ESKD) cases (8). ESKD is an irreversible condition in which the kidneys completely lose the ability to filter waste products and excess fluids (7). As a result, patients become dependent on dialysis or kidney transplantation, while also facing an increased risk of cardiovascular disease and premature death (8).

Stem cell therapy (SCT) is a promising biotechnology technique with wide applications and has made remarkable advances in clinical settings (9). The stem cells that have been used in preclinical and clinical studies include umbilical cord blood mesenchymal stem cells (MSCs) (10), umbilical cord MSCs (11), placental MSCs (12), adipose MSCs (13), and bone marrow mesenchymal stromal cells (BMSCs) (14). Among them, adipose MSCs are the most widely used. Animal experiments have shown that SCT can effectively treat diabetic kidney disease (DKD) (15, 16). However, the safety and efficacy of SCT in patients with DKD remain unknown, and only a few randomized controlled trials (RCTs) with small sample sizes have explored its role in the treatment of patients with DKD. Perico et al. (17) recently conducted a phase 1b/2a multicenter RCT to evaluate the safety, tolerability, and treatment efficacy of adult allogeneic bone marrow stromal stem cell transplantation in patients with moderate-to-severe DKD. The authors found that 18 weeks of SCT resulted in significant improvement of estimated glomerular filtration rate (eGFR) but did not affect the urine microalbumin/creatinine ratio (UACR). However, Gaipov et al. (18) found that SCT significantly reduced microalbuminuria (MAU) without affecting eGFR or serum creatinine (SCr) levels. Therefore, this systematic review and meta-analysis of RCTs aimed to explore the safety and efficacy of SCT in patients with DKD to provide deeper insights into the translation of SCT from clinical trials to the clinical application stages.

## 2 Methods

### 2.1 Protocol and registration

This systematic review and meta-analysis has been registered in PROSPERO (CRD42024520313) and was performed according to the Preferred Reporting Items for Systematic Reviews and Meta-Analyses (PRISMA) guidelines (19).

### 2.2 Search strategy

As of March 4, 2024, two authors (HD and CX) comprehensively retrieved clinical trial data relating to renal-related outcome measures and adverse events in adults with DKD using PubMed, Cochrane Library, Web of Science, and Embase to assess SCT efficacy. The main search terms were “stem cells,” “Diabetic Nephropathies,” “Diabetic Kidney Diseases,” and related keywords. Full details of the retrieval strategy for all the databases can be found in the Supplementary material.

### 2.3 Inclusion criteria

1. Population: Age ≥18 years old; established diagnosis of type 1 or type 2 DM with DKD; eGFR <60 mL/min/1.73 m^2^ for three consecutive months or MAU (albumin of 30–300 mg in a 24-h urine collection). DN was defined as either micro- or macroalbuminuria (albumin >300 mg/24-h) according to the 2007 Kidney Disease Outcomes Quality Initiative Clinical Practice Guidelines and Clinical Practice Recommendations for Diabetes and Chronic Kidney Disease (20). All studies of patients with DKD that reported at least one of the following results were considered for inclusion: UACR, cystatin C, SCr, eGFR, markers of tubular injury, adverse event rate, and mortality.
2. Intervention: Stem cell drug products, regardless of source, type, dose, duration, or route of administration.
3. Comparison intervention: Placebo trials with multiple interventions (e.g., co-administered autologous bone marrow-derived mononuclear cells and umbilical cord-MSCs) were eligible if the study groups differed only in their use of SCs.
4. Outcome(s): The primary outcome was eGFR; and the secondary outcomes were SCr, MAU, UACR, and the incidence of adverse events; other relevant outcome measures included metabolic parameters: hemoglobin A1c, triglycerides, and glucose.
5. Study design (S): RCTS or non-randomized clinical controlled trials (CCTs)

### 2.4 Exclusion criteria

The exclusion criteria were as follows: (1) animal experiments; (2) kidney disease secondary to other diseases; (3) full-text content not available; and (4) missing or duplicated experimental data.

### 2.5 Study selection

After removing duplicate studies, two authors (HD and CX) independently screened all titles and abstracts for potential relevance and acquired the full text of the relevant content. Disagreements were resolved by consensus or by consulting a third author (YY).

### 2.6 Data extraction and literature quality evaluation

#### 2.6.1 Date collection

Two authors (HD and CX) summarized the primary data from the included trials, including the first author and year of publication. If the data were not reported or missing, the corresponding author was emailed. If the authors did not respond, data were obtained from the charts or formulas. Disagreements were resolved by consulting a third author (YY).

#### 2.6.2 Assessment of risk of bias and quality of evidence

The quality of each study included in the analysis was assessed using the Cochrane Risk of Bias Assessment Tool (RevMan 5.40). There were seven items in the bias risk table: (1) random sequence generation (selection bias); (2) allocation concealment (selection bias); (3) blinding of participants and personnel (performance bias); (4) blinding of outcome assessment (detection bias); (5) incomplete outcome data (attrition bias); (6) selective reporting (reporting bias); and (7) other bias. Each item was classified as low risk, high risk (not fulfilling the criteria), or unclear (specific details or descriptions were not reported) (21). Furthermore, the presence of publication bias was estimated using a funnel plot.

### 2.7 Data analysis

Review Manager (5.40, Cochrane Collaboration) software was used for statistical analysis. Between-study heterogeneity was assessed using the Higgins *I*^2^-test. Meaningful heterogeneity was determined at 50% of the *I*^2^ values. Due to significance, a random-effects model was used for the meta-analysis over a fixed-effects model. For dichotomous variable data such as mortality, the risk ratio (RR) and 95% confidence interval (95% CI) were used as the combined effect size estimates. For continuous variables, such as eGFR and SCr, standardized mean difference (SMD) or weighted mean differences and their 95% CI were used as the combined effect size estimates.

## 3 Results

### 3.1 Eligible studies

**Figure 1:**
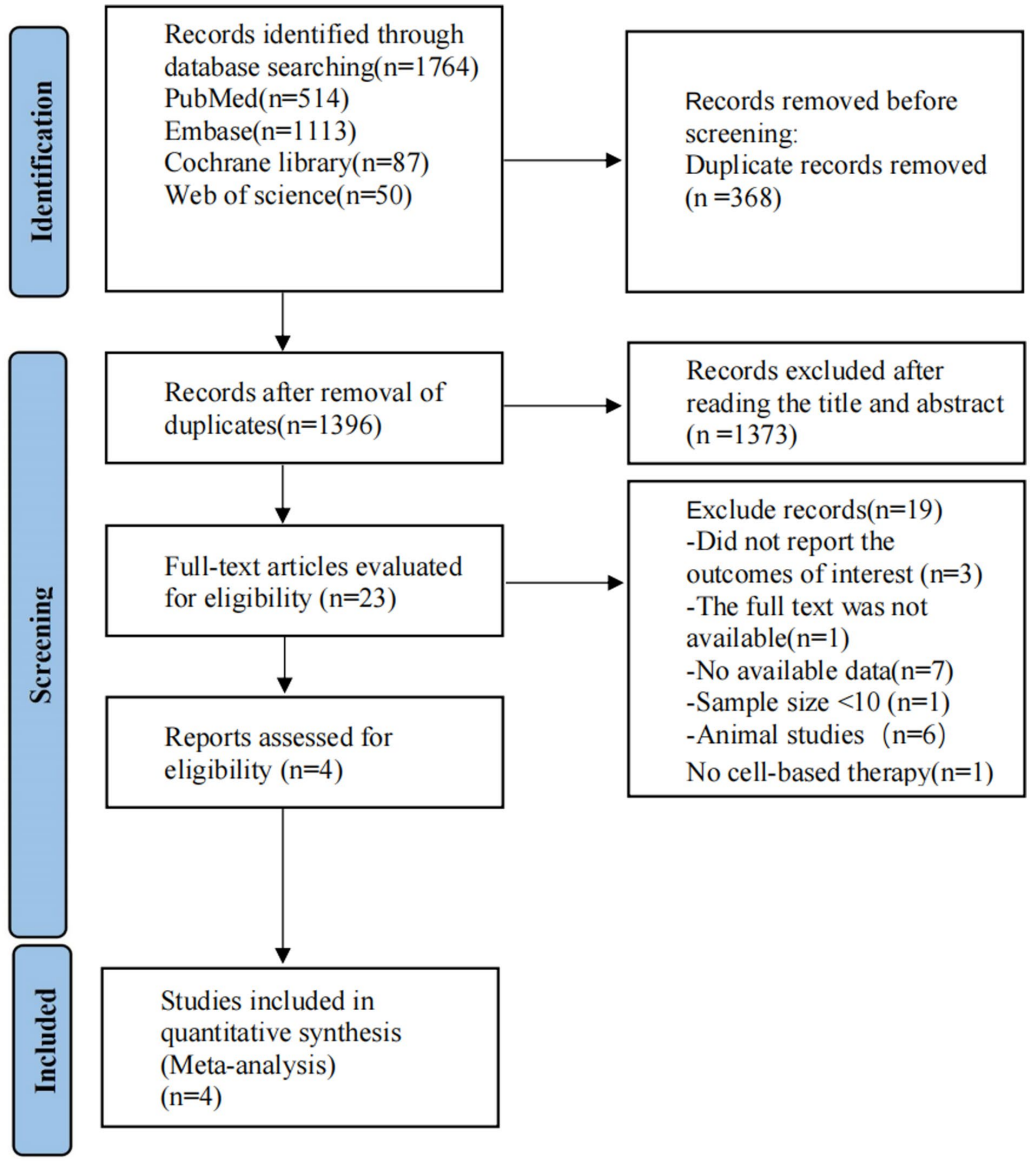
PRISMA flow diagram of the study process. PRISMA, Preferred Reporting Items for Systematic Reviews and Meta-Analyses.

The PRISMA flow diagram is shown in Figure 1. A systematic electronic literature search initially identified 3,528 studies. After applying the exclusion criteria, four trials (17, 18, 22, 23) were included in the meta-analysis.

### 3.2 Study characteristics

Baseline data and interventions are presented in Tables 1, 2. The included studies were published between 2016 and 2023, with four articles and 90 participants. The demographic distribution within the studies is characterized by 60 males (67%) and 30 females (33%), with an average age of approximately 51 years and an age range spanning from 18 to 82 years. A total of 53 (58%) patients underwent SCT. Although SCT was applied in all included studies, the source, dose, frequency, and mode of injection varied. All four studies included used bone marrow as a source of stem cells, and one study used umbilical cord MSCs. Allogeneic administration was employed in two studies, and autologous administration was employed in two studies.

**Table 1:** The characteristics of included participants.

Study	Country	Sample size (*n*)	Disease stage	Age (years)	Sex (M/F)	Stem cell species	Cell purification method	Injection method	Treatment	Control	Treatment-related TEAEs
Perico et al. (17)	Italy	G1: 12	ADN	69.2 ± 2.1	12/0	Allogeneic bone marrow stromal cells	Immunoselection	I.V.	ORBCEL-M	Placebo	Bronchospasm
		CG: 4		59.6 ± 5.7	4/0						
Wu et al. (23)	China	G1: 14	EDN	34.7 ± 5.9	9/6	ABM-MNCs + UC-MSCs	Density gradient centrifugation	I.A.	ABM-MNC	Standard care	None
		CG: 15		35.8 ± 5.5	6/7						
Packham et al. (22)	Australia	G1: 10	ADN	74.8 ± 7.9	8/2	Allogeneic bone-marrow derived MPC	Immunoselection	I.V.	Rexlemestrocel-L	Placebo	None
		G2: 10		70.5 ± 7.4	9/1						
		G3: 10		64.8 ± 10.1	7/3						
Gaipov et al. (18)	Kazakhstan	G1: 8	EDN	26.4 ± 5.4	3/5	ABM-MNCs	Density gradient centrifugation	I.V.	ABM-MNCs	ABM-MNCs	None
		G2: 7		32.4 ± 14.1	2/5						

G1, experimental group; CG, control group; ADN, advanced diabetic nephropathy; EDN, early diabetic nephropathy; MPC, mesenchymal precursor cells; ABM-MNCs, autologous bone marrow-derived mononuclear cells; UC-MSCs, umbilical cord mesenchymal stem cells; I.V., intravenous injection; I.A., arterial injection; TEAEs, treatment-emergent adverse events.

**Table 2:** Study characteristics.

First author, year	Injection dose	Diagnosis	Duration	Primary outcomes	Secondary outcomes
Perico, 2023	80 × 10^6^ cells	T2DM	18 M	Safety: the number and severity of prespecified cell infusion-associated events and the overall number and frequency of AEs and unexpected severe AEs	mGFR, eGFR (MDRD, CKD-EPI), UACR, fasting blood glucose, HbA1c, total cholesterol, LDL cholesterol, triglycerides, BP, anti-HLA antibody development, proportion of total number of circulating lymphocyte (T cells, B cells, and NK cells), myeloid cell (monocytes and dendritic cells) subsets and plasma serum immunoassay-derived concentrations of biomarkers of inflammation
Wu, 2021	1.10 ± 0.22 × 10^6^ MSCs/kg	T1DM	8 Y	The incidence of chronic complications, including DPN, DN, DRP	Safety, HbA1c, exogenous insulin requirement (daily dose), fasting blood glucose, fasting C peptide, microalbumin, SCr, eGFR (MD-RD)
	0.61 ± 0.26 × 10^10^ aBM-MNCs/kg				
Packham, 2016	—	T2DM	60 M	Safety, eGFR (MDRD, CKD-EPI), mGFR	Serum creatinine, creatinine clearance, albumin-creatinine ratio, protein-creatinine ratio, cystatin-C, HbA1c or BP, IL-6, TNF-α, adiponectin, TGF-β, uric acid, FGF23
	150 × 10^6^ cells				
	300 × 10^6^ cells				
Gaipov, 2018	140 × 10^6^ cells	T1DM		NGAL, Urinary type-IV collagen, microalbuminuria, eGFR (CKD-EPI)	Fasting C-peptide, fasting serum insulin, HbA1C, glucose fasting, glucose postprandial, insulin-replacement, insulin short-acting, insulin long-acting, β-blockers, ACE-inhibitors, Ca-channel blockers

T1DM, type 1 diabetes mellitus; T2DM, type 1 diabetes mellitus; AE, adverse event; M, months; Y, years; mGFR, measured glomerular filtration rate; eGFR, estimated glomerular filtration rate; MDRD, modification of diet in renal disease; CKD-EPI, Chronic Kidney Disease Epidemiology Collaboration; UACR, urine albumin/creatinine ratio; HbA1c, hemoglobin A1c; LDL, low-density lipoprotein; BP, blood pressure; DN, diabetic nephropathy; SCr, serum creatinine; IL, interleukin; TNF, tumor necrosis factor; TGF, transforming growth factor; HLA, human leukocyte antigen; DPN, diabetic peripheral neuropathy; DRP, diabetic retinopathy; FGF23, fibroblast growth factor 23; NGAL, neutrophil gelatinase-associated lipocalin; ACE, angiotensin-converting enzyme.

### 3.3 Quality assessment of the articles

**Figure 2:**
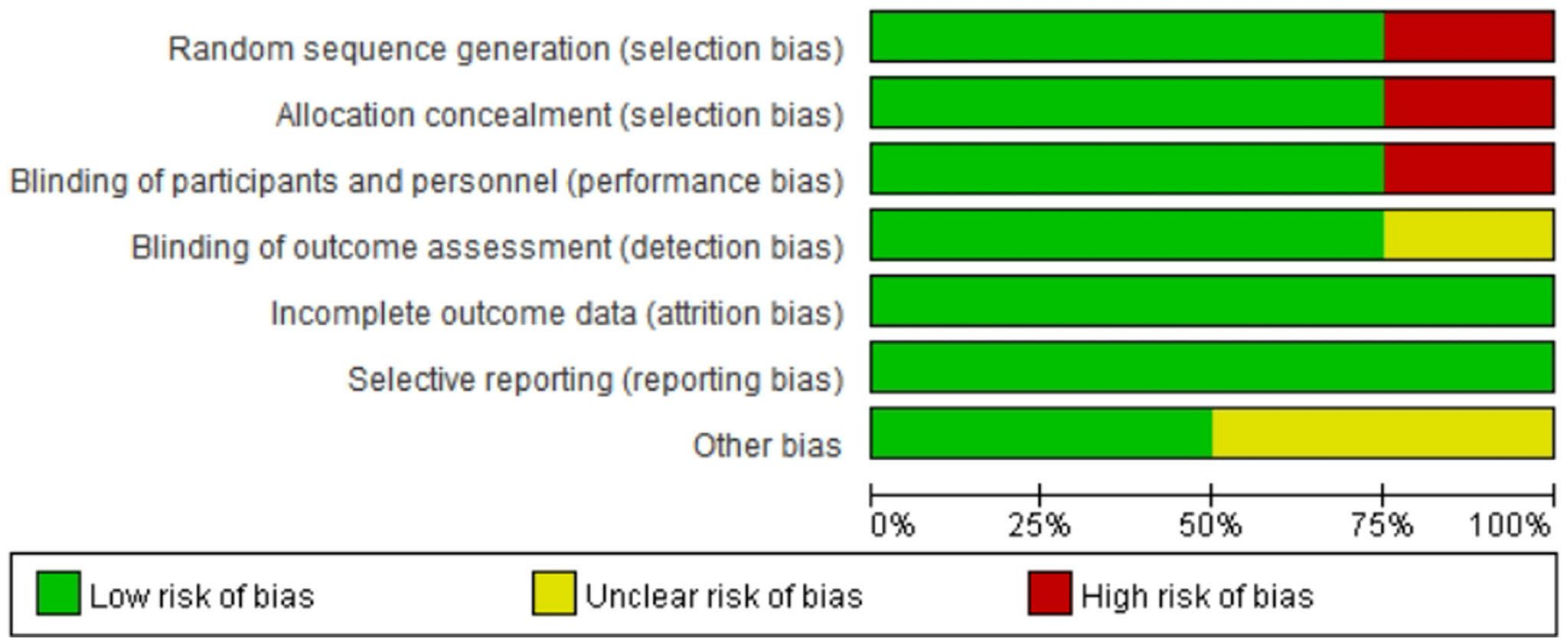
Risk of bias graph: judgments of each risk of bias item, presented as a percentage.

**Figure 3:**
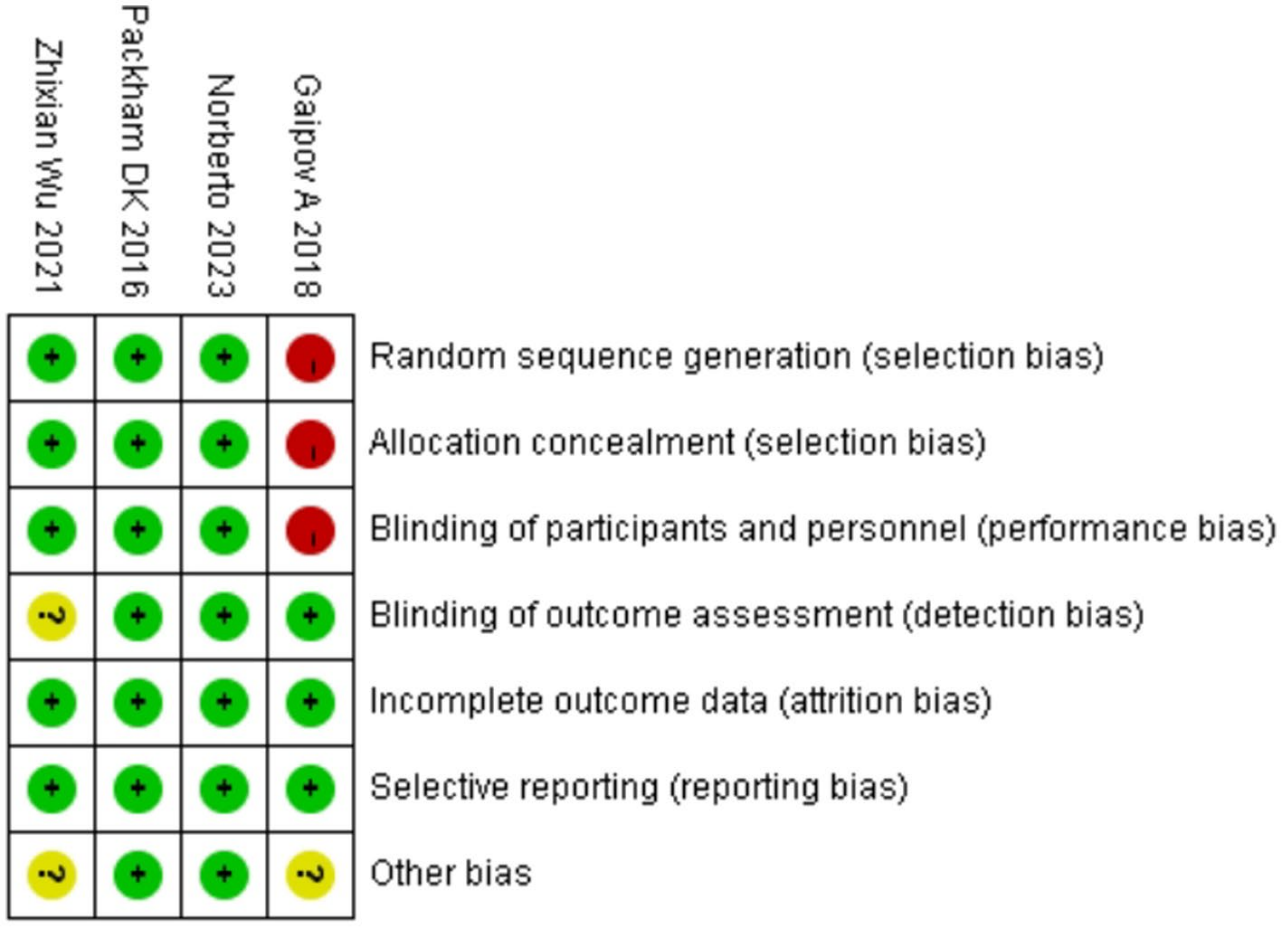
Risk of bias summary: judgments of each risk of bias item for each included study.

Figures 2, 3 summarize the risk of bias in the included studies. The four studies had different study designs; three studies were RCTs (17, 18, 22), and one was a prospective, open-label study (23). Furthermore, quality assessment of these studies revealed that three studies had a low risk of bias (17, 18, 22), and one study had an unclear to high risk of bias, as its investigators did not apply the blinding procedure rationally (23). Overall, the included RCTs had a low risk of bias.

### 3.4 Outcome

#### 3.4.1 Effect of SCT on eGFR

**Figure 4:**
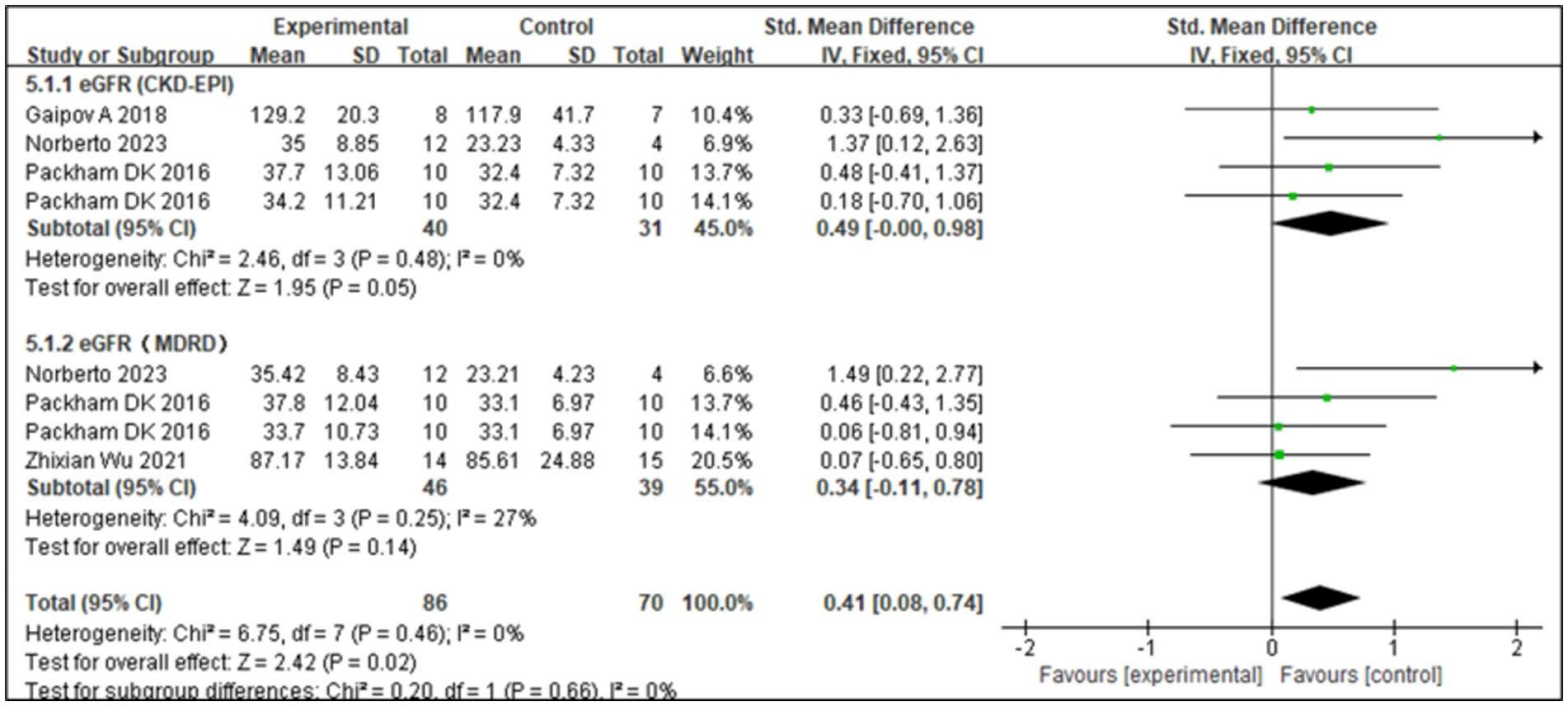
Subgroup analysis for estimated glomerular filtration rate (eGFR).

**Figure 5:**
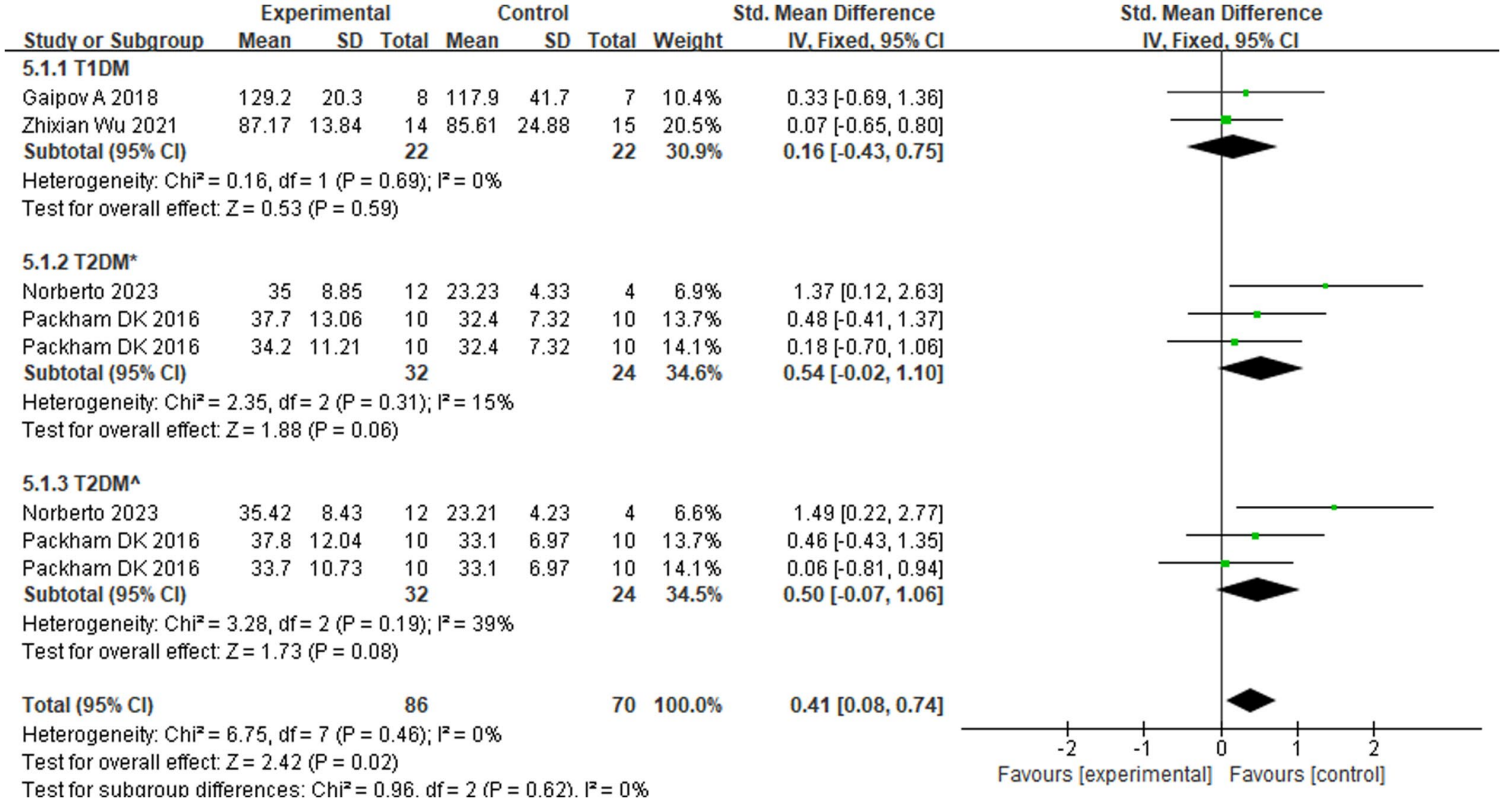
Subgroup analysis for eGFR in different types of diabetes. T2DM^*^: eGFR was measured according to the Chronic Kidney Disease Epidemiology Collaboration (CKD-EPI) formula. T2DM^^^: eGFR was measured according to the Modification of Diet in Renal Disease (MDRD) formula.

The eGFR is an important indicator of renal function. The Modification of Diet in Renal Disease and Chronic Kidney Disease Epidemiology Collaboration formulas are commonly used to estimate GFR. Four studies (17, 22, 23) showed that SCT significantly improved eGFR levels (*Z* = 3.56; *p* = 0.02). Analysis of forest plot data (Figure 4) showed significant improvement with SCT as the intervention, compared with the outcome in the control group (MD = 0.41, 95% CI: 0.08–0.74; *p* = 0.025). Two of the included studies focused on patients with T1DM, while the other two involved patients with T2DM. Given the distinct pathogenic mechanisms, there is an inherent age variation among the study groups. To more precisely assess the efficacy of SCT in enhancing eGFR, a subgroup analysis was conducted. The *I*^2^ value was 0%, indicating no heterogeneity, thus a fixed-effects model was used. The pooled analysis (Figure 5) reveals that SCT exerted a significant therapeutic effect on eGFR across DKD patients with varying diabetes sub-types (SMD = 0.41, 95% CI: 0.08–0.74; *p* = 0.02).

#### 3.4.2 Effect of SCT on SCr

**Figure 6:**
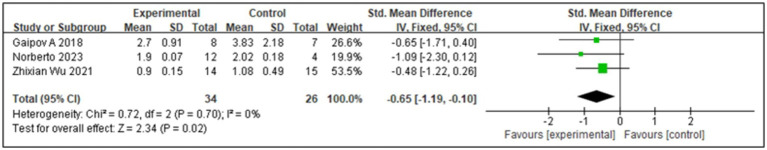
Forest plot for serum creatinine (SCr).

SCr levels can be used to monitor DKD. In the early stages of DKD, SCr may remain within the normal range, but its levels gradually increase with disease progression; therefore, monitoring SCr levels is important for the early diagnosis and disease monitoring of DKD. Three studies (17, 18, 23) reported SCr levels, and the associated *I*^2^ value was 0%. Therefore, we used a fixed-effects model in this study. The results from the forest plot analysis (Figure 6) showed that treatment with SCT was associated with significant changes in SCr levels (*Z* = 2.34; *p* = 0.02), and the trial group with stem cell injection as the intervention showed significantly reduced SCr levels in patients with diabetes (SMD = −0.65, 95% CI = −1.19 to −0.1, *p* = 0.02).

#### 3.4.3 Effect of SCT on MAU

**Figure 7:**
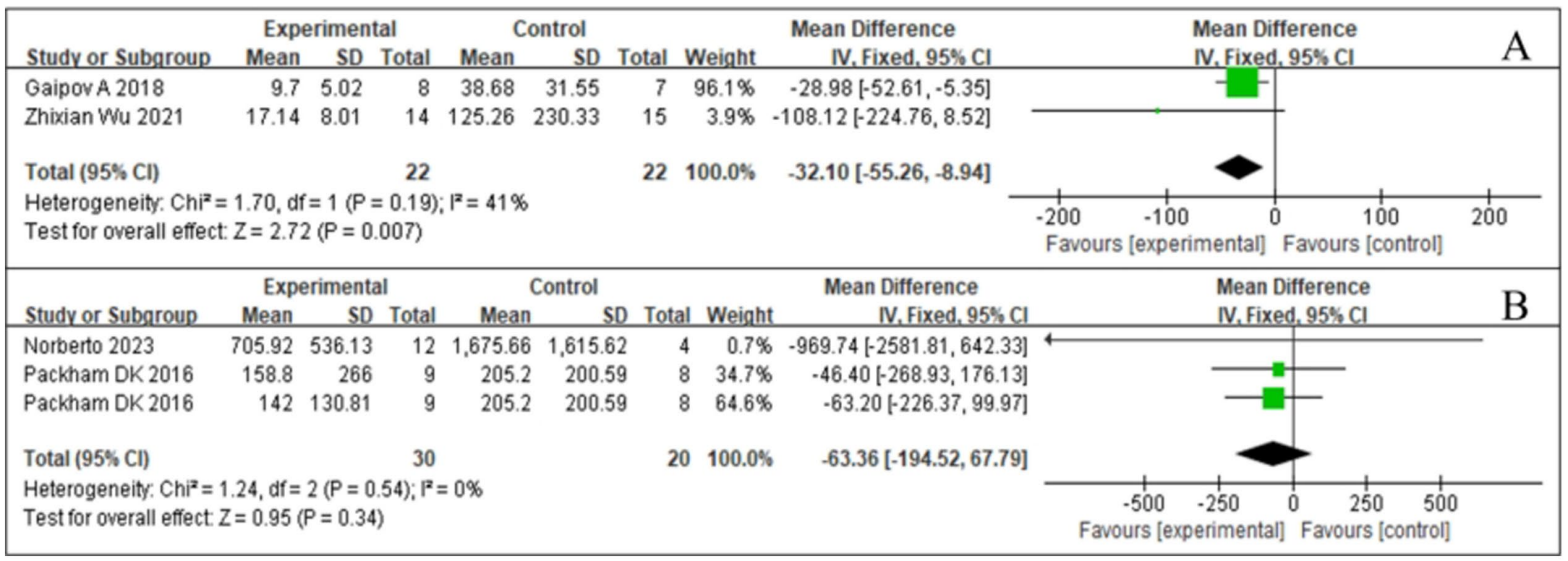
Forest plot for urine markers. **(A)** Microalbuminuria (MAU). **(B)** Urine albumin/creatinine ratio (UACR).

MAU is an early hallmark of DKD. Persistent MAU was significantly positively associated with the risk of developing clinical proteinuria in patients with diabetes, indicating that MAU is important for preventing DKD development. A comprehensive analysis of MAU was conducted in two studies (18, 23), presented in Figure 7A. SCT was associated with significant changes in MAU levels (*Z* = 2.72; *p* = 0.007), with low inter-study heterogeneity and an *I*^2^ value of 41, suggesting high agreement between the findings. Analysis of the forest plot data showed that MAU levels in the SCT group were significantly lower than those in the control group (MD = −32.1, 95% CI = −55.26 to −8.94, *p* = 0.007).

#### 3.4.4 Effect of SCT on UACR

UACR is an indicator for urinary protein excretion and is a key parameter in the early screening of DKD. Elevated UACR predicts the presence of kidney injury, especially in patients with diabetes. Integrating the available data (18, 23), we visually demonstrated the results of the UACR study (Figure 7B). After statistical analysis, the effect of SCT in reducing UACR did not meet the requirements of statistical significance (*Z* = 0.95, *p* = 0.34). In addition, inter-study heterogeneity was low (*I*^2^ = 0%, *p* = 0.54). No significant difference in the UACR was found between the test and control groups (MD = −63.36, 95% CI: −194.52 to 67.79, *p* = 0.34) (see Figure 7B).

#### 3.4.5 Adverse event reporting results

We performed a meta-analysis of the studies (17, 22, 23) with respect to the SCT-induced AEs in Table 3. The AEs were categorized into eight major systems: circulatory, respiratory, digestive, nervous, urinary, musculoskeletal, endocrine, and immune systems, as well as unclassified events. The experimental group reported 67 AEs in 60 subjects. The control group reported 35 AEs in 28 subjects. Combined, there were 102 AEs in 88 subjects across all systems. The highest number of AEs was observed in the respiratory system, with 29 events in 22 subjects. The experimental group had 15 events in 12 subjects, while the control group had 14 events in 10 subjects. This suggests a similar incidence of respiratory AEs in both groups. The second-highest number of AEs was observed in the endocrine system, with 16 events in 13 subjects. The experimental group had 9 events in 8 subjects, while the control group had 7 events in 5 subjects. Notably, severe hypoglycemia accounted for a significant portion of these events. In the urinary system, the experimental group reported 4 events in 4 subjects, while the control group had no AEs in this category. This highlights a potential safety concern specific to the experimental intervention. Meanwhile, in the nervous system, the experimental group had a higher number of events (9 in 9 subjects) compared to the control group (1 in 1 subject), indicating a possible increased risk of neurological AEs with the experimental intervention. The specific classification of AEs can be found in Supplementary Table 1.

**Table 3:** Summary of adverse event.

System	Experimental group total (cases/subjects)	Control group total (cases/subjects)
Circulatory system	11/8	3/2
Respiratory system	15/12	14/10
Digestive system	7/7	3/3
Nervous system	9/9	1/1
Urinary system	4/4	0/0
Musculoskeletal system	4/4	4/4
Endocrine system	9/8	7/5
Immune system	3/3	2/2
Unclassified	5/5	1/1
Total	67/60	35/28

## 4 Discussion

A previous systematic review demonstrated the significant effect of SCT on chronic kidney disease in animal models by showing that it can help reduce the incidence of DKD. This treatment effectively improved kidney function while reducing the release of kidney injury markers, renal fibrosis, and inflammatory mediators, as well as high glucose levels, MAU, eGFR, and SCr levels (15, 16, 24). Previous studies have largely been based on these models; however, the efficacy and safety of SCT for DKD remain nebulous owing to the lack of long-term clinical trial data. In particular, the types of stem cells, their sources, and the selection of dosages are controversial among different studies. In this study, we included four RCTs and found that SCT safely and effectively improved eGFR and SCr levels and reduced MAU in patients with DKD. However, SCT did not improve UACR or blood sugar levels (Supplementary Figure S1). Additionally, there was no significant difference in the incidence of adverse events between the two groups.

The efficacy of SCT through various cell delivery pathways and in various cell types remains controversial. Intravenous delivery of MSCs, currently the most widely studied cell type for DKD and related kidney diseases, is restricted by the lungs and spleen, which results in a low number of cells reaching the kidney that may not be sufficiently active (25). Following the intravenous infusion, most MSCs remain in the lungs in the short term, with 50–60% of MSCs remaining in the lungs at 1 h post-injection, decreasing to 30% after 3 h, and maintaining stable levels at 96 h (26). Subsequently, the MSCs are gradually cleared from the lungs and accumulate in the liver and spleen. This phenomenon is known as the “lung first-pass effect” (27). Due to their large size, MSCs are easily trapped in the lung capillaries. Therefore, different infusion routes or preconditioning methods may increase the number and activity of MSCs reaching the kidney.

Other types of cells may have better results in improving kidney outcomes. For example, UC/AF cells reduce SCr, fibrosis, and inflammation similar to MSCs and to a greater extent than by non-MSCs (28). Compared to MSCs, UC/AF cells also reduced proteinuria to a greater extent. Arterial injection can avoid pulmonary entrapment in the first cycle and improve the targeting efficiency. Researchers have examined the efficacy of various cell delivery pathways in animal models of chronic kidney disease. In a meta-analysis, the caudal vein (70% of studies, 28 animals) was the most effective in reducing renal function outcomes; however, in one study, renal artery delivery was more effective in reducing anti-fibrotic factors than previously reported. Rashed et al. (29) and Han et al. (30) have shown that melatonin (MT) preconditioning can improve the proliferative antioxidant capacity and angiogenesis capacity of BMSCs and enhance their therapeutic effect on DN by promoting the recovery of neurotrophic effects and myelination. These methods may increase the accumulation of MSCs in the kidneys, thereby enhancing their therapeutic effect. In our meta-analysis, all cells were MSCs, and only one of the included studies (23) used arterial injections. However, there was no significant difference in SCr or eGFR levels between the SCT and control groups, unlike in MAU levels. Future studies may provide a clear answer regarding the superior cell injection pathways and cell tissue sources in DKD therapy.

The two most effective biomarkers for assessing kidney health are eGFR and albuminuria (or proteinuria) (31). eGFR is the gold standard for accurately measuring overall kidney function (32). In addition, estimates of eGFR are based on serological biomarkers of renal filtration, most commonly SCr (33). In existing animal models and clinical trials, SCT is associated with improvements in renal function, such as stabilization or enhancement of GFR and reduction of proteinuria. Lin et al. (15) in a meta-analysis, found that SCT has a potential renoprotective effect, significantly reducing SCr and blood urea nitrogen levels and mitigating renal impairment. The meta-analysis by Papazova et al. (16) showed that SCT could reduce the occurrence and progression of chronic kidney disease, especially through the improvement of urinary protein, SCr, and eGFR levels. The results of the meta-analysis in this study are consistent with these findings, showing that SCT significantly improved the degree of disease activity, albuminuria, SCr, and eGFR levels in DKD. However, the GFR level at which individuals benefit the most from SCT remains undetermined, and this “treatment window” has been explored in clinical nephrology trials, including the angiotensin-receptor blocker irbesartan in DN (34) and fish oil in IgA nephropathy (35).

In addition, we found differences in the efficacy of cell therapies at the molecular level as well as changes in blood glucose levels between different species, which may be related to the tightly controlled conditions and detailed evaluation of animal trials. Clinical trials must consider more practical application factors, such as individual differences and concomitant diseases. For example, Ezquer et al. (36) administered pluripotent mesenchymal stromal cells to mice with DM to study the preventive effect of SCT on chronic kidney disease secondary to DM; this led to the regeneration of the pancreas and kidneys by reversing high blood sugar levels and reducing proteinuria. In another study, Ezquer et al. (37) observed a reduction in proteinuria despite hyperglycemia and hypoinsulinemia following transplantation of autologous bone marrow mesenchymal stem cells (AB-MSCs), highlighting the direct renoprotective function of stem cells. The opposite results were obtained by Liu et al. (38). DN was induced in Sprague–Dawley rats using intrabitoneal injection of streptozotocin, and, after MSC transplantation, the blood glucose level showed improvements but proteinuria did not improve. Wang et al. (39) investigated direct renal regeneration in experimental rat models with type 1 DN, where intra-arterial administration of BMSCs prevented the development of proteinuria and podocyte damage or loss but did not improve blood glucose levels. In the current meta-analysis, SCT treatment was significantly effective in reducing albuminuria but not in improving glycemic control in patients with DKD. This result should be interpreted with caution as it is based on pooled data from a small number of studies.

MSC infusion reduces the production of profibrotic markers and inflammatory factors, as demonstrated by decreased levels of interleukin (IL)-6 and tumor necrosis factor-α (TNF-α) and increased levels of the anti-inflammatory cytokines IL-4 and IL-10 (40, 41). Li et al. (26) determined the levels of validated cytokines in serum samples of DN rats using Milliplex rat cytokine kit and suggested that MSC treatment significantly reduced the expression of IL-1α, IL-1β, IL-6, and interferon-*γ*. After lipopolysaccharide stimulation of macrophages, the expression of proinflammatory cytokines such as IL-6, IL-1β, TNF-α, and monocyte chemoattractant protein-1 increased. SCT can also serve as treatment for other kidney diseases. Chang et al. (42) evaluated the role of MSCs in anti-Thy1.1-induced glomerulonephritis rat models and found that the intrarenal transplantation of hypoxia-preconditioned MSCs reduced glomerular apoptosis, autophagy, and inflammation. Song et al. (43) in adriamycin (ADR) nephropathy rats, showed that MSCs reduced oxidative stress and inflammation by inhibiting nuclear factor-kappa B and improved glomerular sclerosis and interstitial fibrosis, alleviating ADR nephropathy. In the clinical trials included in this study, SCT did not have a prominent anti-inflammatory effect; Perico’s et al. trial (17) showed an increasing trend in the serum inflammatory biomarkers such as soluble TNF receptor 1, neutrophil gelatinase-associated lipocalin, and vascular cell adhesion molecule 1 during the 18-month follow-up period, with no difference between groups. A multicenter RCT study by Packham et al. (22) showed no significant change in TNF-α levels. Owing to the differences in the anti-inflammatory effects of MSCs observed in animal models and clinical trials, the inflammatory markers selected in different studies may differ, and the measurement methods may affect the interpretation of the results. For example, some studies may use more sensitive biomarkers or more precise measurement techniques that more accurately reflect changes in the inflammatory status. In animal studies, the route of administration of MSCs (intravenous injection and intrarenal transplantation) and dosage may differ from those in clinical trials. In humans, higher doses of MSCs or specific routes of administration may be required to achieve anti-inflammatory effects similar to those observed in animals.

Exploring the potential mechanisms underlying cell-based regenerative therapies is key in treating DKD. MSCs protect the kidneys from damage through multiple pathways involving autonomously targeted, anti-apoptotic, anti-inflammatory, antioxidant, and anti-fibrotic effects and podocyte autophagy regulation (44, 45) The mechanism of this therapy is mainly achieved through two pathways: the paracrine action of stem cells and the exosomes secreted by stem cells (24, 46). First, MSCs reduce the expression of transforming growth factor β1 (TGFβ1) and inhibit the transdifferentiation of glomerular cells into myofibroblasts, which is a key pathological process in renal fibrosis. In addition, MSCs reduce the abnormal proliferation of glomerular cells by inhibiting the activation of phosphatidylinositol 3-kinase/Akt and mitogen-activated protein kinase signaling pathways, which are key factors in extracellular matrix (ECM) accumulation and glomerular expansion in DN. MSCs can also increase the expression of matrix metalloprotein 2 (MMP2) and MMP9, promote the degradation of ECM proteins, and reduce excessive accumulation of ECM. Simultaneously, MSCs secrete various cell growth factors, such as epidermal growth factor, which reduce the apoptosis of podocytes induced by hyperglycemia and promote the repair and regeneration of podocytes. Second, stem cells play a therapeutic role by secreting exosomes. Exosomes contain a variety of microRNAs (miRNAs) and mRNAs that regulate gene expression in target cells. For example, miR-21 inhibits the expression of programmed cell death protein 4 and reduces TGF-β-induced fibrosis. miR-192 and miR-215 downregulate E-cadherin expression and alleviate renal fibrosis. Exosomes transfer their contents to damaged tissues, promote the proliferation of glomerular and tubular epithelial cells, inhibit apoptosis, and repair damaged kidney tissues. Exosomes also inhibit the inflammatory response, reduce the infiltration of inflammatory cells and the production of inflammatory factors, and reduce the inflammation of the glomeruli and renal tubules (47–49).

The main challenges in applying SCT in patients with DKD are efficacy. T1DM is an autoimmune disease characterized by the destruction of pancreatic β-cells, leading to an absolute deficiency of insulin. Exogenous insulin therapy is particularly crucial in T1DM (50). Meanwhile, many T2DM patients eventually require exogenous insulin therapy as the disease progresses (51). SCT that aim to improve insulin independence at the source represent an innovative approach in the treatment strategies for diabetes. The latest clinical trial (NCT04786262) has showcased a groundbreaking advancement in the treatment of T1DM with VX-880. VX-880, an allogeneic stem cell therapy, has the capability to differentiate into islet cells. When administered via the hepatic portal vein, it homes to the liver and commences insulin secretion. Following a single, full-dose infusion of VX-880, all T1DM patients successfully received islet cell transplantation. Notably, most significantly reduced or even completely eliminated the need for exogenous insulin. The SCT has also shown breakthroughs in the management of T2DM and its complications, especially in promoting pancreatic regeneration and reducing insulin resistance (52). A patient with ESKD had autologous MSCs transdifferentiated into induced pluripotent stem cells (iPSCs) and received regenerative islet transplantation. The patient successfully got rid of the dependence on exogenous insulin after surgery, and the oral hypoglycemic drugs were gradually discontinued. The kidney and other indicators were normal, indicating that SCT effectively prevented the deterioration of complications. The emergence of SCT signifies a revolutionary shift in the therapeutic strategies for diabetes, offering the potential for physiological reconstruction of islet function. Our research findings indicate that SCT has shown promise and efficacy in controlling markers of kidney damage, yet there is a necessity for further studies to explore its applicability across various subtypes of diabetes and to address the limitations inherent in current research.

Exosomes are cell-secreted nanovesicles that naturally contain biomolecular cargoes such as lipids, proteins, and nucleic acids (53). They function as intercellular communicators, transporting a diverse cargo of bioactive molecules—including proteins, lipids, messenger RNAs (mRNAs), and microRNAs (miRNAs)—from the parent MSCs to recipient cells (54). This cargo endows exosomes with inherent therapeutic properties relevant to DKD, demonstrating remarkable efficacy in treatment. Relevant research suggests that through exosomal delivery of miRNA-16-5p or miRNA-26a-5p, can protect podocytes from hyperglycemia-induced damage (55). Chronic inflammation is a cornerstone of DKD progression (56). Exosomes could be engineered to deliver anti-inflammatory cytokines like IL-10 directly to the inflamed renal microenvironment. For instance, adipose-derived MSC (ADMSC) exosomes have been shown to suppress IL-6 production in glomerular mesangial cells via miR-125a, thereby mitigating mesangial hyperplasia and kidney fibrosis (56). While the prospect of using engineered stem cell derivatives for DKD appears promising, the long-term stability of the final engineered exosome product also needs to be ensured. Moreover, advanced engineered therapies will require even more rigorous validation processes.

In conclusion, while the SCT may offer therapeutic benefits, the safety profile, as indicated by the higher incidence of AEs, cannot be overlooked. The meta-analysis reveals that the experimental intervention is associated with a higher overall frequency of AEs compared to the control group. Specifically, the experimental group reported 67 AEs in 60 subjects, while the control group reported 35 AEs in 28 subjects. This suggests that the experimental intervention may carry an increased risk of adverse outcomes, which warrants careful consideration. The distribution of AEs across different systems provides further granularity into the potential risks associated with the experimental intervention. Notably, the respiratory system and endocrine system exhibited the highest incidence of AEs, with 29 events in 22 subjects and 16 events in 13 subjects, respectively. In the respiratory system, the experimental group reported 15 events in 12 subjects, compared to 14 events in 10 subjects in the control group. This indicates a similar incidence rate between the groups but highlights the need for vigilance in monitoring respiratory-related AEs. In contrast, the endocrine system showed a more pronounced difference, with the experimental group experiencing 9 events in 8 subjects, compared to 7 events in 5 subjects in the control group. This discrepancy is particularly concerning given the potential long term implications of endocrine-related AEs, such as severe hypoglycemia. Another noteworthy finding is the complete absence of AEs in the urinary system within the control group, compared to 4 events in 4 subjects in the experimental group. This stark contrast suggests a potential intervention-related risk that requires further investigation. The presence of such events in the experimental group alone raises attention about the safety profile of the intervention and its impact on renal function and overall urinary health. The potential intervention-related adverse events in the urinary system highlight the need for long-term follow-up studies to assess the chronic effects on renal function. The nervous system also exhibited a higher incidence of AEs in the experimental group, with 9 events in 9 subjects, compared to only 1 event in 1 subject in the control group. This suggests that the experimental intervention may have neurotoxic effects or other neurological implications that need to be explored in future studies. Additionally, the musculoskeletal system showed a balanced distribution of AEs, with 4 events in 4 subjects in both groups, indicating that the intervention may not significantly impact this system. Future research should prioritize targeted monitoring and mechanistic studies to address these safety concerns, and conduct long-term follow-up to ensure that the intervention can be safely implemented in clinical practice.

### 4.1 Limitations

The study had several limitations. First, the number of RCTs included in this study was small, possibly contributing to the risk of not accounting for all findings. Second, most clinical stem cell studies are still in early phases, with significant variations in stem cell isolation, purification methods, and injection routes. Critically, the reporting of detailed stem cell collection, apheresis, and processing protocols within the included primary studies is often inconsistent and incomplete. These variations in product collection and preparation techniques can significantly impact the composition and potency of the administered cell product. This issue was not explicitly addressed in our analysis and likely contributes to the heterogeneity in outcomes. This highlights the current lack of standardized, effective strategies for precisely targeting stem cells to damaged tissues in clinical practice. Different transplantation methods demonstrably impact MSC survival and homing rates; consequently, the optimal implantation technique, treatment timing, and number of injections remain to be determined. Third, due to the limited number and small sample size of the included RCTs, we were unable to validate the treatment effects at the level of circulating cytokines or other mechanistic biomarkers, which are crucial for understanding the biological pathways involved. Fourth, the analysis relied solely on trial-level data, incorporating only main trial results. Individual patient data were unavailable; such data could clarify whether stem cell benefits are specific to DKD patients. While our subgroup analysis used eGFR, its reliability will improve with greater access to individual patient data. Fifth, the progression from DKD to ESKD varies widely among patients (taking years to decades), influenced by baseline renal function, glycemic control, blood pressure management, and lifestyle. Trial participants were at different DKD stages during follow-up, likely leading to varied responses to cell therapy. This heterogeneity inevitably introduces bias into the meta-analysis results. Although the topic remains controversial, stem cell therapy for DKD is a promising field—provided it is appropriately and thoroughly addressed. Therefore, future studies must prioritize larger-scale randomized controlled trials involving participants at similar stages of kidney injury, rigorously standardizing and reporting cell product characteristics, incorporating mechanistic biomarker assessments to verify our conclusions and advance the field reliably.

## 5 Conclusion

The results of this study suggest that SCT can serve as a potential treatment modality for DKD and that it can significantly improve eGFR, decrease SCr, and reduce MAU, thus reducing renal damage. However, this study also showed that SCT was not effective in improving UACR levels. Owing to the obvious heterogeneity between the included studies, our results should be verified in RCTs with large sample sizes.

## Data Availability

The original contributions presented in the study are included in the article/Supplementary material, further inquiries can be directed to the corresponding author.
