# Supplementary material for: Efficacy of stem cell therapy for diabetic kidney disease: a systematic review and meta-analysis
Source: Front Med (Lausanne). 2025 Sep 1;12:1601900. doi: 10.3389/fmed.2025.1601900 (PMC12433957; doi:10.3389/fmed.2025.1601900)
Supplement: Supplementary file 1 [file Data_Sheet_1.docx]

**Search Strategies for All Databases**

**PubMed:514**

#1：**("Diabetic Nephropathies"[Mesh]) OR (((((((((((((((((Nephropathies, Diabetic) OR (Nephropathy, Diabetic)) OR (Diabetic Nephropathy)) OR (Diabetic Kidney Disease)) OR (Diabetic Kidney Diseases)) OR (Kidney Disease, Diabetic)) OR (Kidney Diseases, Diabetic)) OR (Diabetic Glomerulosclerosis)) OR (Glomerulosclerosis, Diabetic)) OR (Intracapillary Glomerulosclerosis)) OR (Nodular Glomerulosclerosis)) OR (Glomerulosclerosis, Nodular)) OR (Kimmelstiel-Wilson Syndrome)) OR (Kimmelstiel Wilson Syndrome)) OR (Syndrome, Kimmelstiel-Wilson)) OR (Kimmelstiel-Wilson Disease)) OR (Kimmelstiel Wilson Disease))**

#2：**(((((((((((((((("Stem Cells"[Mesh]) ) OR (Cell, Stem)) OR (Cells, Stem)) OR (Stem Cell)) OR (Progenitor Cells)) OR (Cell, Progenitor)) OR (Cells, Progenitor)) OR (Progenitor Cell)) OR (Mother Cells)) OR (Cell, Mother)) OR (Cells, Mother)) OR (Mother Cell)) OR (Colony-Forming Unit)) OR (Colony Forming Unit)) OR (Colony-Forming Units)) OR (Colony Forming Units)**

#3：#1 **OR** #2

"Diabetic Nephropathies"[MeSH Terms] OR ("Diabetic Nephropathies"[MeSH Terms] OR ("diabetic"[All Fields] AND "nephropathies"[All Fields]) OR "Diabetic Nephropathies"[All Fields] OR ("nephropathies"[All Fields] AND "diabetic"[All Fields]) OR "nephropathies diabetic"[All Fields] OR ("Diabetic Nephropathies"[MeSH Terms] OR ("diabetic"[All Fields] AND "nephropathies"[All Fields]) OR "Diabetic Nephropathies"[All Fields] OR ("nephropathy"[All Fields] AND "diabetic"[All Fields]) OR "nephropathy diabetic"[All Fields]) OR ("Diabetic Nephropathies"[MeSH Terms] OR ("diabetic"[All Fields] AND "nephropathies"[All Fields]) OR "Diabetic Nephropathies"[All Fields] OR ("diabetic"[All Fields] AND "nephropathy"[All Fields]) OR "diabetic nephropathy"[All Fields]) OR ("Diabetic Nephropathies"[MeSH Terms] OR ("diabetic"[All Fields] AND "nephropathies"[All Fields]) OR "Diabetic Nephropathies"[All Fields] OR ("diabetic"[All Fields] AND "kidney"[All Fields] AND "disease"[All Fields]) OR "diabetic kidney disease"[All Fields]) OR ("Diabetic Nephropathies"[MeSH Terms] OR ("diabetic"[All Fields] AND "nephropathies"[All Fields]) OR "Diabetic Nephropathies"[All Fields] OR ("diabetic"[All Fields] AND "kidney"[All Fields] AND "diseases"[All Fields]) OR "diabetic kidney diseases"[All Fields]) OR ("Diabetic Nephropathies"[MeSH Terms] OR ("diabetic"[All Fields] AND "nephropathies"[All Fields]) OR "Diabetic Nephropathies"[All Fields] OR ("kidney"[All Fields] AND "disease"[All Fields] AND "diabetic"[All Fields]) OR "kidney disease diabetic"[All Fields]) OR ("Diabetic Nephropathies"[MeSH Terms] OR ("diabetic"[All Fields] AND "nephropathies"[All Fields]) OR "Diabetic Nephropathies"[All Fields] OR ("kidney"[All Fields] AND "diseases"[All Fields] AND "diabetic"[All Fields]) OR "kidney diseases diabetic"[All Fields]) OR ("Diabetic Nephropathies"[MeSH Terms] OR ("diabetic"[All Fields] AND "nephropathies"[All Fields]) OR "Diabetic Nephropathies"[All Fields] OR ("diabetic"[All Fields] AND "glomerulosclerosis"[All Fields]) OR "diabetic glomerulosclerosis"[All Fields]) OR ("Diabetic Nephropathies"[MeSH Terms] OR ("diabetic"[All Fields] AND "nephropathies"[All Fields]) OR "Diabetic Nephropathies"[All Fields] OR ("glomerulosclerosis"[All Fields] AND "diabetic"[All Fields]) OR "glomerulosclerosis diabetic"[All Fields]) OR ("Diabetic Nephropathies"[MeSH Terms] OR ("diabetic"[All Fields] AND "nephropathies"[All Fields]) OR "Diabetic Nephropathies"[All Fields] OR ("intracapillary"[All Fields] AND "glomerulosclerosis"[All Fields]) OR "intracapillary glomerulosclerosis"[All Fields]) OR ("Diabetic Nephropathies"[MeSH Terms] OR ("diabetic"[All Fields] AND "nephropathies"[All Fields]) OR "Diabetic Nephropathies"[All Fields] OR ("nodular"[All Fields] AND "glomerulosclerosis"[All Fields]) OR "nodular glomerulosclerosis"[All Fields]) OR ("Diabetic Nephropathies"[MeSH Terms] OR ("diabetic"[All Fields] AND "nephropathies"[All Fields]) OR "Diabetic Nephropathies"[All Fields] OR ("glomerulosclerosis"[All Fields] AND "nodular"[All Fields]) OR "glomerulosclerosis nodular"[All Fields]) OR ("Diabetic Nephropathies"[MeSH Terms] OR ("diabetic"[All Fields] AND "nephropathies"[All Fields]) OR "Diabetic Nephropathies"[All Fields] OR ("kimmelstiel"[All Fields] AND "wilson"[All Fields] AND "syndrome"[All Fields]) OR "kimmelstiel wilson syndrome"[All Fields]) OR ("Diabetic Nephropathies"[MeSH Terms] OR ("diabetic"[All Fields] AND "nephropathies"[All Fields]) OR "Diabetic Nephropathies"[All Fields] OR ("kimmelstiel"[All Fields] AND "wilson"[All Fields] AND "syndrome"[All Fields]) OR "kimmelstiel wilson syndrome"[All Fields]) OR ("Diabetic Nephropathies"[MeSH Terms] OR ("diabetic"[All Fields] AND "nephropathies"[All Fields]) OR "Diabetic Nephropathies"[All Fields] OR ("syndrome"[All Fields] AND "kimmelstiel"[All Fields] AND "wilson"[All Fields])) OR ("Diabetic Nephropathies"[MeSH Terms] OR ("diabetic"[All Fields] AND "nephropathies"[All Fields]) OR "Diabetic Nephropathies"[All Fields] OR ("kimmelstiel"[All Fields] AND "wilson"[All Fields] AND "disease"[All Fields]) OR "kimmelstiel wilson disease"[All Fields]) OR ("Diabetic Nephropathies"[MeSH Terms] OR ("diabetic"[All Fields] AND "nephropathies"[All Fields]) OR "Diabetic Nephropathies"[All Fields] OR ("kimmelstiel"[All Fields] AND "wilson"[All Fields] AND "disease"[All Fields]) OR "kimmelstiel wilson disease"[All Fields]))

**Embase:1113**

#1：**('diabetic nephropathies'/exp OR 'diabetic nephropathies' OR (('diabetic'/exp OR diabetic) AND nephropathies) OR (nephropathies, AND diabetic) OR (nephropathy, AND diabetic) OR (diabetic AND nephropathy) OR (diabetic AND kidney AND disease)) AND diabetic AND kidney AND diseases OR (kidney AND disease, AND diabetic) OR (kidney AND diseases, AND diabetic) OR (diabetic AND glomerulosclerosis) OR (glomerulosclerosis, AND diabetic) OR (intracapillary AND glomerulosclerosis) OR (nodular AND glomerulosclerosis) OR (glomerulosclerosis, AND nodular) OR ('kimmelstiel wilson' AND syndrome) OR (kimmelstiel AND wilson AND syndrome) OR (syndrome, AND 'kimmelstiel wilson') OR ('kimmelstiel wilson' AND disease) OR (kimmelstiel AND wilson AND disease)**

#2：**.'stem cells'/exp OR 'stem cells' OR (('stem'/exp OR stem) AND ('cells'/exp OR cells)) OR (cell, AND stem) OR (cells, AND stem) OR (stem AND cell) OR (progenitor AND cells) OR (cell, AND progenitor) OR (cells, AND progenitor) OR (progenitor AND cell) OR (mother AND cells) OR (cell, AND mother) OR (cells, AND mother) OR (mother AND cell) OR ('colony forming' AND unit) OR (colony AND forming AND unit) OR ('colony forming' AND units) OR (colony AND forming AND units)**

#3：#1 **and** #2

**Cochrane Library:87**

#1：**MeSH descriptor: [Diabetic Nephropathiesl explode all trees**

#2：**(Diabetic Nephropathies or Nephropathies, Diabeticor Nephropathy, Diabetic or Diabetic Nephropathy or Diabetic Kidney Disease or Diabetic kidneyDiseases or Kidney Disease, Diabetic or Kidney Diseases, Diabetic or Diabetic Glomerulosclerosis or Glomerulosclerosis, Diabetic or intracapiaryGlomerulosclerosis or Nodular Glomerulosclerosis or Glomerulosclerosis, Nodular or Kimmelstiel.Wilson Syndrome or Kimmelstiel Wilson Syndrome or Syndrome, Kimmelstiel-Wilson or Kimmelstiel-Wilson Disease or Kimmelstiel Wilson Disease):ti,ab,kw**

**(Osteoarthrosis):ti,ab,kw OR (Osteoarthroses):ti,ab,kw**

#3：#1 **or** #2

#4：**MeSH descriptor: [Stem Cells] explode all trees**

#5：**(Stem Cells or Cell, Stem or Cels, Stem or Stem Cell or Progentor Cells or Cel, Progenitor or Cels, Progenitor or Progenitor Cell or Mother Cells or Cel,Mother or Cels, Mother or Mother Cell or colony.Forming Unit or Colony Forming Unit or Colony-Forming Units or Colony Forming Units):t,ab,kw**

#6：#4 **or** #5

#7：#3 **and** #6

**Web of Science:50**

#1：**Stem Cells (Topic) or Cell, Stem (Topic) or Cells, Stem (Topic) or Stem Cell (Topic) or Progenitor Cells (Topic) or Cell, Progenitor (Topic) or Cells, Progenitor (Topic) or Progenitor Cell (Topic) or Mother Cells (Topic) or Cell, Mother (Topic) or Cells, Mother (Topic) or Mother Cell (Topic) or Colony-Forming Unit (Topic) or Colony Forming Unit (Topic) or Colony-Forming Units (Topic) or Colony Forming Units (Topic)**

#2：**Diabetic Nephropathies (Topic) or Nephropathies, Diabetic (Topic) or Nephropathy, Diabetic (Topic) or Diabetic Nephropathy (Topic) or Diabetic Kidney Disease (Topic) or Diabetic Kidney Diseases (Topic) or Kidney Disease, Diabetic (Topic) or Kidney Diseases, Diabetic (Topic) or Diabetic Glomerulosclerosis (Topic) or Glomerulosclerosis, Diabetic (Topic) or Intracapillary Glomerulosclerosis (Topic) or Nodular Glomerulosclerosis (Topic) or Glomerulosclerosis, Nodular (Topic) or Kimmelstiel-Wilson Syndrome (Topic) or Kimmelstiel Wilson Syndrome (Topic) or Syndrome, Kimmelstiel-Wilson (Topic) or Kimmelstiel-Wilson Disease (Topic) or Kimmelstiel Wilson Disease (Topic)**

#3：#1 **and** #2
