# Supplementary figures and images for: Efficacy of stem cell therapy for diabetic kidney disease: a systematic review and meta-analysis
Source: Front Med (Lausanne). 2025 Sep 1;12:1601900. doi: 10.3389/fmed.2025.1601900 (PMC12433957; doi:10.3389/fmed.2025.1601900)

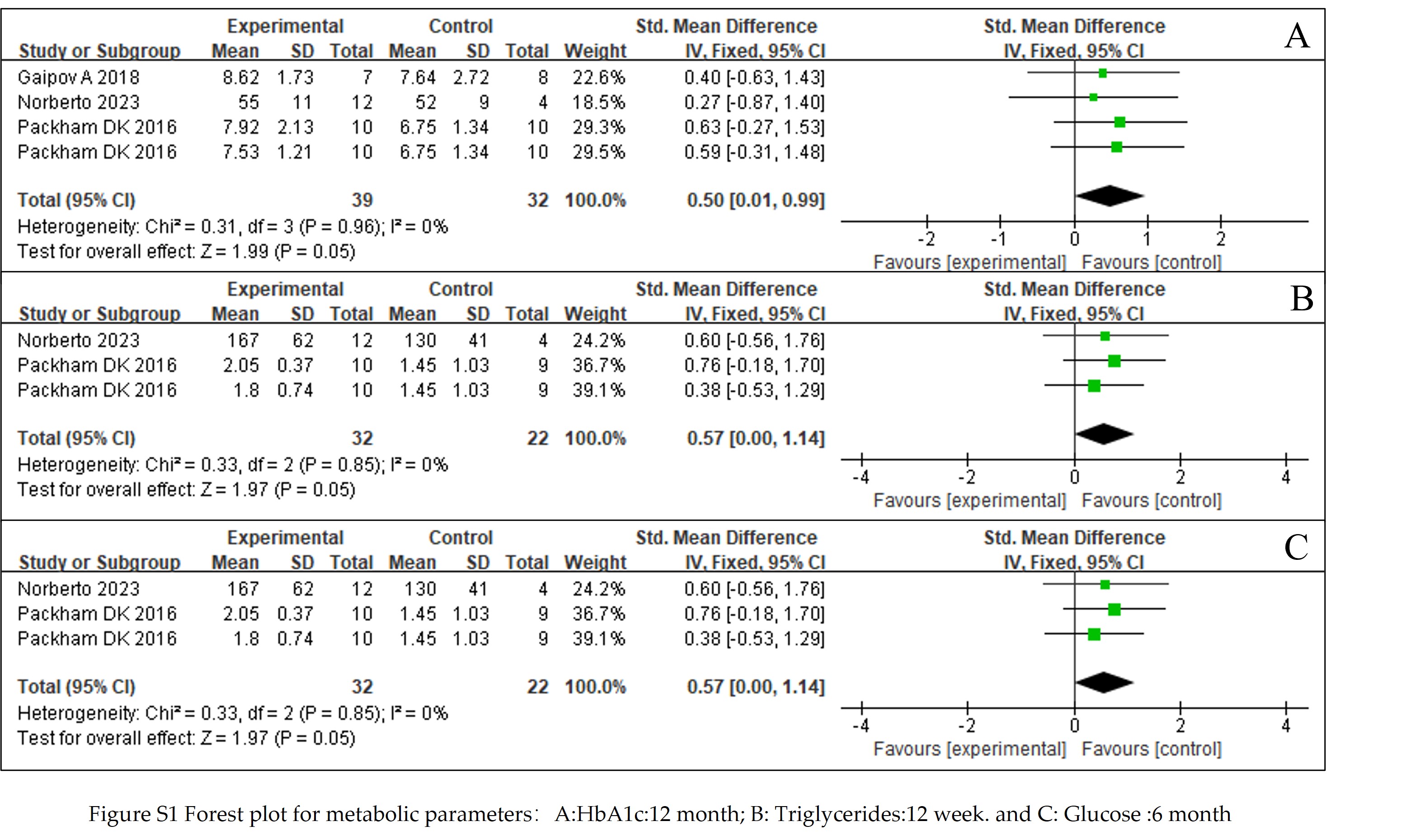

Supplement: Supplementary file 2 [file Image_1.jpeg]
